# Diagnosis of cardiac occupancy as metachronous carcinoma using multimodal imaging: a case report

**DOI:** 10.1186/s12872-022-02645-2

**Published:** 2022-05-04

**Authors:** Zihan Wang, Xiang Xiao, Shuying Lv, Chunyan Li, Hong Jiang

**Affiliations:** 1 Beijing University of Chinese Medicine, Beijing, People’s Republic of China; 2 Department of Integrative Cardiology, China-Japan Friendship Hospital, Beijing, People’s Republic of China

**Keywords:** Cardiac malignancy, Metachronous carcinoma, Multimodal imaging, Case report

## Abstract

**Background:**

Metachronous carcinoma presenting as a cardiac malignancy is rare, and timely diagnosis is critical. We report a patient with a primary cardiac tumor who eventually died and performed an imaging-related literature review.

**Case presentation:**

A 68-year-old Chinese male patient, who had suffered from multiple malignancies, was suddenly found to have severely reduced platelets and symptoms of decreased cardiac function. After undergoing a series of imaging examinations such as transthoracic echocardiography and positron emission tomography-computed tomography, he was found to have a large occupancy within the right heart and was finally diagnosed with a primary cardiac malignancy. Combined with the patient's previous medical history, it was judged that this time it was a metachronous carcinoma. The patient was unable to accept the risk of surgery and eventually died.

**Conclusion:**

This is a case report reporting a cardiac malignancy. This case highlights the importance of using multiple imaging modalities to make a common diagnosis and the need for more detailed evaluation in patients with metachronous carcinoma.

**Supplementary Information:**

The online version contains supplementary material available at 10.1186/s12872-022-02645-2.

## Background

Multiple primary cancers presenting as primary malignant tumor of the heart have rarely been reported. In addition, sudden findings of thrombocytopenia with coagulation dysfunction are predictive of adverse events. Here, we report a case of a cardiac tumor diagnosed as multiple primary cancers by multimodality cardiac imaging. Also, we review and summarize the literature on multiple primary cancers and related aspects of cardiac imaging diagnosis.

## Case presentation

A 68-year-old male patient was admitted to the hospital on 2019-10-02 with the symptoms of "chest tightness, shortness of breath, and weakness with panic for 4 months and reduced platelets for 2 months". He began experiencing chest tightness and shortness of breath with panic and weakness 4 months ago, however, he was tested only 2 months ago and was found to have a low platelet count of 30*10^9^/L. He was treated with oral Amino-polypeptide only thereafter.

The patient was diagnosed with "laryngeal carcinoma" in 2016 and the pathology showed "highly differentiated squamous cell carcinoma T3N2M0, stage IVA", which was surgically removed after one cycle of chemotherapy with TPF regimen (Paclitaxel, Nedaplatin, Tegafur) in combination with Nimotuzumab. In 2017, a new primary "esophageal carcinoma" appeared and pathology showed "intermediate differentiated squamous carcinoma", and esophagectomy was performed. 2018, a metastatic tumor in the left cervical lymph node appeared which was diagnosed as "squamous cell carcinoma" and underwent lymph node dissection. After this, the patient had regular follow-up outpatient visits every 3–6 months, had several imaging examinations, and the echocardiography, electrocardiogram, and chest computed tomography reports of the last 1 year did not reveal any significant cardiopulmonary abnormalities, d-dimer was always < 0.5 mg/L, pulmonary function and oxygen saturation were always good, and the rest of the blood tests were normal, with stable vital signs and no evidence suggesting tumor recurrence. He was never found to have diabetes or cardiovascular disease, and had no specific family history of genetic disease. The patient had smoked 20 cigarettes per day for the past 45 years and had quit for 6 months, and had drunk 150 ml of alcohol per day for the past 30 years and had quit for 3 years.

The patient had bilateral lower extremity edema and was admitted with blood pressure of 135/90 mmHg. Blood tests revealed hemoglobin (HGB) of 126 g/L, platelets (PLT) of 34 × 10^9^/L, d-dimer (D-D) of 20 mg/L, prothrombin time (PT) of 17.9 s, activated partial thromboplastin time (APTT) of 46.0 s, fibrinogen quantitation (Fib) of 1.72 g/L, N-terminal pro brain natriuretic peptide (NT-proBNP) of 2448 pg/mL. The patient was seen in a state of persistent low platelets and disorders of coagulation factors during hospitalization. Tumor marker assay showed neuronspecific enolase (NSE) of 32 ng/ml, serum osteopontin (CYFRA21-1) of 29.83 ng/ml, serum CA125 of 120.4U/ml, and squamous cell carcinoma antigen (SCC) of 2.85 ng/ml. The peripheral blood smear showed normal leukocytes, varying size of mature erythrocytes, the presence of poikilocyte and orthochromatic normoblast, and a significant decrease in platelets. Bone marrow aspiration (flow cytometric immunophenotyping) showed 6.28% lymphocytes, 45.6% CD5 + cells, 64.27% myeloid cells, normal percentage of CD10 + mature granulocytes; 1.59% monocytes, high percentage of CD56 + cells (39.81%), 15.26% nucleated red blood cells, diminished CD36 expression and abnormal phenotype; 0.13% CD34 + , CD117 + naive myeloid cells, the proportion was not high and the phenotype was not abnormal; CD19 + B progenitor cells within CD34 + cells accounted for 5.94%, the proportion was approximately normal; the phenotype of the red lineage was mildly abnormal. In addition, all antibodies associated with autoimmune diseases were negative.

**Fig. 1:**
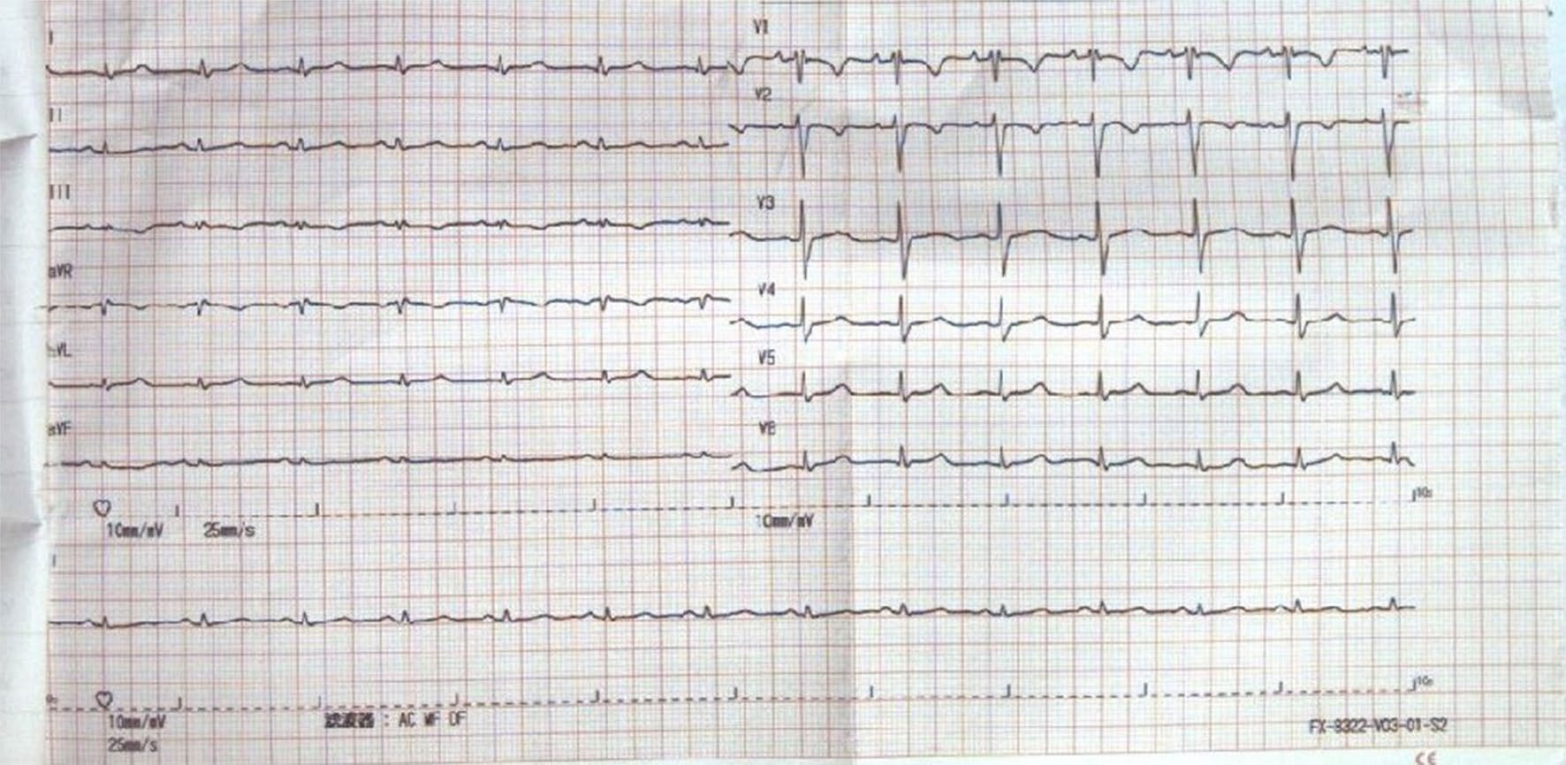
Electrocardiogram showed low potentials, nothing unusual

**Fig. 2:**
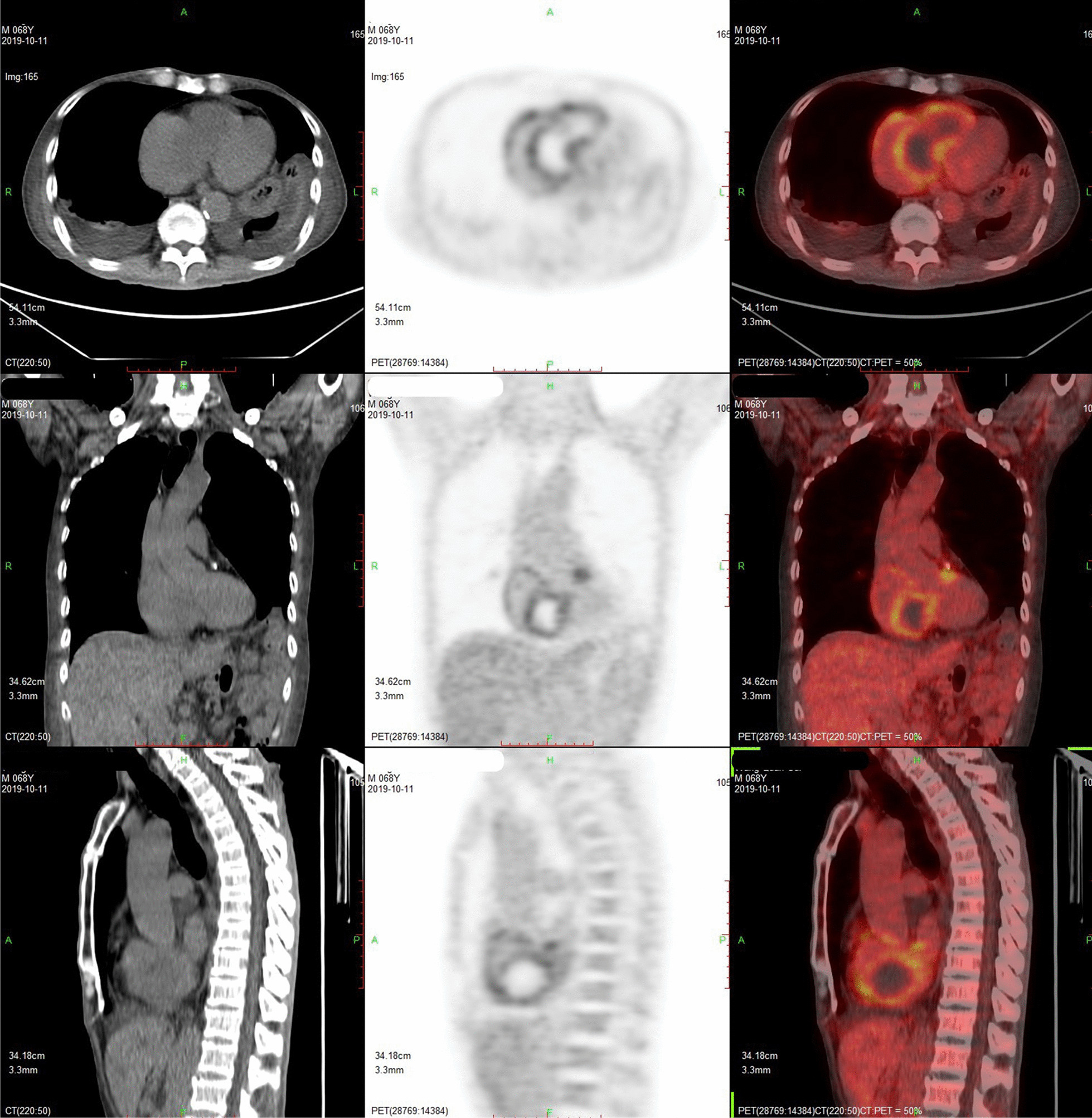
PET-CT results showed that the right heart was enlarged, and hypointense shadow was seen in the right ventricle, right atrium and the beginning segment of the main pulmonary artery, with a ring-shaped radioactive inhomogeneous thickening, ring-shaped increased glucose metabolism, and the hypointense shadow within it was a radioactive sparse defect area, considering a possible primary malignant lesion of the heart. Scattered lamellar hyperintensities in both lungs and a left lower lobe striate shadow with increased glucose metabolism were considered inflammatory lesions. The right hilar lymph node with increased glucose metabolism was considered reactive hyperplasia

No specific changes were seen on the electrocardiogram (Fig. 1). Transthoracic echocardiography (TTE) (Additional file 1: Video 1) suggested the presence of an occupancy in the patient's right heart cavity with a range of approximately 73*34 mm. Echocardiography and contrast echocardiography (Additional file 2: Video 2, Additional file 3: Video 3) showed the presence of a 76*40 mm mass in the right atrium and right ventricle with abundant blood supply in the mass and a possible regional necrotic area near the right ventricular outflow tract. Computed tomography pulmonary angiography (CTPA) showed multiple mass filling defects in the right atrium, right ventricle, and right ventricular outflow tract, and multiple lobar segmental pulmonary emboli in both lungs. Positron emission tomography-computed tomography (PET-CT) (Fig. 2) showed a suspected primary malignant tumor of the heart.

The patient had multiple previous tumors, elevated tumor markers, echocardiography revealed the presence of an occupancy with abundant internal blood flow, and PET-CT showed only high glucose metabolism within the right heart occupancy, and no hypermetabolic cancerous tissue was observed in the intrapulmonary vessels. In the more than 1 year since the last tumor, no further tumor was detected and the imaging after admission showed a single lesion. After a multidisciplinary discussion, we diagnosed the patient with a metachronous carcinoma in the right heart. Based on the progression of the disease, we also believe that the malignant cardiac tumor led to the development of disseminated intravascular coagulation (DIC) and thrombocytopenia, and that a new pulmonary embolism developed as the tumor progressed. The final treatment was resection of the mass. However, patients with low platelets and gradually decreasing oxygen saturation are extremely weak and cannot tolerate any surgical procedures or anti-tumor treatments, so he can only be treated mainly with supportive therapy, strengthening intravenous nutrition and receiving human albumin, propyloglobulin, platelets and plasma transfusions. The patient's pulmonary embolism complicated by thrombocytopenia limited the use of anticoagulant medications, and his condition continued to deteriorate over a 2-week period, culminating in his death from cardiogenic shock. Due to lack of consent, we did not perform an autopsy.

## Discussion

Multiple primary cancers are defined as the synchronous (synchronous carcinoma, SC) or metachronous (metachronous carcinoma, MC) occurrence of more than one cancer in the same individual. Multiple primary cancers are mostly seen in people around 70 years old, and the peak incidence is concentrated within 5–10 years of the first primary cancer. According to the chronological order of appearance, if different tumors appear more than 6 months apart, like this patient, it is considered as MC [1]. According to the International Association of Cancer Registries and the International Agency for Research on Cancer, the overall frequency of multiple primary cancers varies between 2.4 and 17% [2]. Due to the rising incidence of cancer, the long-term side effects of chemotherapy and radiation therapy, increased diagnostic sensitivity, and the continued influence of genetic and behavioral risk factors, on the other hand, advances in cancer treatment have improved the survival rate of cancer patients, the above has increased the risk of multiple primary cancers. However, MC with cardiac tumors as the first symptom is extremely rare.

Treatment for multiple primary cancers is often individualized according to the type of tumor. When conditions are right, surgical resection is often the best approach, or a combination of therapies such as concurrent chemoradiotherapy (CRT) [3]. Treatment should first deal with lesions that have poor prognosis, rapid progression, and can be directly life-threatening. It has been proposed [4] that staged treatment can reduce the invasiveness of surgery or the toxicity of CRT, but there is also a risk of second tumor progression while treating the first tumor. Patients with multiple cancers have complex conditions. Can they tolerate the side effects of simultaneous treatment of multiple tumors? Should they be given different treatment regimens when multiple tumors are combined? However, there is a lack of in-depth clinical studies and no authoritative guidelines.

Most cardiac tumors are asymptomatic or present with mild and atypical symptoms, and most are at an advanced stage at the time of diagnosis [5, 6]. The differential diagnosis of cardiac masses is crucial for the clinical management of patients because of their diversity (clots, bulky masses, primary or metastatic malignancies) and there are no guidelines or consensus statements on the best diagnostic or therapeutic approach globally. Diagnosis of cardiac tumors first requires assessment of the patient's medical history, whether primary benign or secondary malignant tumors can cause myocardial or valvular dysfunction, which can be accompanied by symptoms of heart failure (most commonly dyspnea), angina pectoris, syncope and electrocardiographic disturbances, and even lethal arrhythmias, often accompanied by weight loss and fatigue, with tumor granule embolism being a more common complication [7, 8].

Primary cardiac tumors are extremely rare, they are mostly benign, about a quarter are malignant, and sarcomas account for 95% of these malignant tumors [9]. In the past, cardiac tumors were diagnosed by histological specimens examined after surgery or death, however, the current rapid development of multimodal cardiac imaging techniques can be applied to the vast majority of diagnoses. Transthoracic echocardiography (TTE), or transesophageal echocardiography (TEE), is the easiest diagnostic method to provide accurate information on tumor morphology, localization and movement, and ultimately to assess its hemodynamic impact. Cardiac imaging methods also include cardiac magnetic resonance (CMR), cardiac computed tomography (CT), and PET-CT, which complement and enhance the evaluation of cardiac masses [10]. Surgical resection is the best treatment modality for cardiac tumors [9].

In this case, TTE determined the location and morphology of the intracardiac mass. After enhancement with ultrasound agents, we evaluated the location and morphology of the mass in more detail, obtaining additional morphological features and important information about tumor vascular perfusion [11, 12] and hemodynamic effects, angiogenesis was observed within the mass, and finally the right atrial and right ventricular occupancies were judged to be tumorigenic rather than thrombotic based on the abundance of blood flow.

The patient had elevated D-D, clinical manifestations of dyspnea and right ventricular failure, and we applied for CTPA. CT can provide information on the distribution of tumor vessels, the degree of tumor calcification, the presence of adipose tissue, and the stage of the disease [13, 14]. The results of CTPA showed pulmonary embolism, while providing precise histological features of intracavitary occupancy in the right heart, considering a tumorigenic occupancy. Given that PET-CT is the most effective method to help in the definitive diagnosis of tumors and differentiate benign from malignant, and to predict survival [10, 15], we performed a whole-body scan using PET-CT, which suggested a "large cardiac primary tumor" located in the right heart cavity, originating in the right atrium and right ventricle of the heart, with the highest probability of malignancy, and showed no signs of metastases. The patient was unable to lie down and was extremely weak, so myocardial and mass biopsies could not be performed to determine the histological nature. Angiosarcoma is mostly found in the right atrium, while rhabdomyosarcoma and fibrosarcoma are located in the ventricle [16], so after multidisciplinary consultation, the patient was finally diagnosed with "a large primary malignant tumor of the heart (located in the right heart cavity and right ventricular outflow tract, the nature of the tumor may be angiosarcoma), pulmonary embolism, heart failure (right heart failure), secondary thrombocytopenia". We conducted a meticulous evaluation and repeated argumentation in order to restore the entire course of the disease and finally concluded with these diagnoses, which, however, may be very close to the truth but still have some uncertainty because of the lack of clear histological findings.

This patient had previous primary laryngeal cancer, esophageal cancer, and this rediagnosis of cardiac tumor with an onset interval longer than 6 months, so the diagnosis of metachronous carcinoma was made. The case report suggests that the appearance of a system-related symptom without an obvious cause or the sudden onset of coagulation system abnormalities strongly suggests the risk of tumor progression or multiple carcinomas, and we clinicians need to develop a close follow-up plan for patients with multiple carcinomas, use multimodal imaging techniques for differential diagnosis of tumors, and develop appropriate treatment measures in a timely manner.

## Supplementary Information

**Additional file 1**. Video 1: TTE showed an occupancy of about 73*34 mm in the right heart cavity.

**Additional file 2**. Video 2: Echocardiography showed a right atrial and intraventricular mass of 76*40 mm.

**Additional file 3**. Video 3: Contract echocardiography showed a rich blood supply and a possible regional necrotic area near the right ventricular outflow tract.

## Data Availability

Some, not all, original data may be able to be shown upon request, to a limited extent. However, some other data cannot be done so due to the confidentiality of the patients’ personal information.

## Abbreviations

SC: Synchronous carcinoma
MC: Metachronous carcinoma
TTE: Transthoracic echocardiography
CTPA: Computed tomography pulmonary angiography
PET-CT: Positron emission tomography-computed tomography
CT: Computed tomography
CRT: Concurrent chemoradiotherapy
DIC: Disseminated intravascular coagulation
